# Preparation of Homopolymer, Block Copolymer, and Patterned Brushes Bearing Thiophene and Acetylene Groups Using Microliter Volumes of Reaction Mixtures

**DOI:** 10.3390/polym13244458

**Published:** 2021-12-19

**Authors:** Joanna Smenda, Karol Wolski, Kamila Chajec, Szczepan Zapotoczny

**Affiliations:** Faculty of Chemistry, Jagiellonian University, Gronostajowa 2, 30-387 Krakow, Poland; joanna.smenda@doctoral.uj.edu.pl (J.S.); kamila.chajec@doctoral.uj.edu.pl (K.C.); zapotocz@chemia.uj.edu.pl (S.Z.)

**Keywords:** polymer brushes, metal-free ATRP, conjugated polymers, patterned films, atomic force microscopy, reversible deactivation radical polymerization, block copolymer brushes

## Abstract

The synthesis of surface-grafted polymers with variable functionality requires the careful selection of polymerization methods that also enable spatially controlled grafting, which is crucial for the fabrication of, e.g., nano (micro) sensor or nanoelectronic devices. The development of versatile, simple, economical, and eco-friendly synthetic strategies is important for scaling up the production of such polymer brushes. We have recently shown that poly (3-methylthienyl methacrylate) (PMTM) and poly (3-trimethylsilyl-2-propynyl methacrylate) (PTPM) brushes with pendant thiophene and acetylene groups, respectively, could be used for the production of ladder-like conjugated brushes that are potentially useful in the mentioned applications. However, the previously developed syntheses of such brushes required the use of high volumes of reagents, elevated temperature, or high energy UV-B light. Therefore, we present here visible light-promoted metal-free surface-initiated ATRP (metal-free SI-ATRP) that allows the economical synthesis of PMTM and PTPM brushes utilizing only microliter volumes of reaction mixtures. The versatility of this approach was shown by the formation of homopolymers but also the block copolymer conjugated brushes (PMTM and PTPM blocks in both sequences) and patterned films using TEM grids serving as photomasks. A simple reaction setup with only a monomer, solvent, commercially available organic photocatalyst, and initiator decorated substrate makes the synthesis of these complex polymer structures achievable for non-experts and ready for scaling up.

## 1. Introduction

Surface-initiated reversible deactivation radical polymerizations (SI-RDRPs) serve as powerful synthetic techniques enabling the decoration of various organic and inorganic substrates by functional polymer brushes via the “grafting from” approach [1]. Surface-initiated atom transfer radical polymerization (SI-ATRP) is the most popular method among SI-RDRPs due to its well-described mechanism, compatibility with various monomers and substrates, as well as commercial availability of necessary reagents [2]. Furthermore, this method allows the formation of polymer brushes with controllable thickness and various architectures, such as homopolymer [3], block copolymer [4], loop [5], mixed [6] or ladder-like brushes [7]. Classical SI-ATRP requires the utilization of high amounts of copper-based catalyst, which is unfavorable in the case of biomedical and electronic applications of the brushes. In response to that, many researchers have moved their attention to SI-ATRP with a ppm concentration of copper catalyst [8,9] or SI-ATRP catalyzed by less harmful metal catalysts [10]. Moreover, recent progress in SI-RDRPs resulted in the development of photoinduced SI-ATRP, which may not only use low concentrations of metal-based catalyst [11,12] but also enables polymer brush synthesis using microliter volumes of reaction mixture [13]. Photoinduced SI-ATRP is a very convenient method for the production of patterned brushes [11,14] that are commonly produced by complex techniques, such as electron-beam [15] and dip-pen lithography [16,17], a combination of photolithography and etching [18,19], or microcontact printing [20]. It is worth emphasizing that patterned brushes or polymer films are of great interest due to their potential applications, such as platforms for the selective adsorption of biomolecules, cell culture substrates, sensors, information storage devices, or nano/microelectronics [21,22]. However, the utilization of metal-based SI-ATRP methods could still be problematic in the case of monomers that can form complexes with metals or undergo other side reactions with a catalyst [23].

The complete elimination of metal ions was achieved in metal-free SI-ATRP catalyzed by organic photocatalysts [24]. As recently reported by the Hawker group, this method could be used for the production of patterned brushes with various functionality using only microliter volumes of reagents under ambient conditions [25]. Moreover, we have shown that pyridine-based monomers that are difficult to polymerize by classical ATRP due to their high affinity to form complexes with metals could be easily polymerized by metal-free SI-ATRP [26]. Therefore, this powerful technique is convenient for obtaining polymer brushes with various functional groups. 

One very interesting class of surface-grafted polymers is conjugated polymer brushes (CPBs), which could be produced by the “grafting to” or “grafting from” approach. Surface-grafted CPBs with high grafting density enable the formation of platforms of molecular wires oriented orthogonally with respect to the modified surface, showing anisotropic properties potentially favorable for nanoelectronics applications and photovoltaic solar cells [27]. CPBs were used as efficient hole transporting layers in organic solar cells replacing PEDOT:PSS [28,29]. Furthermore, Kiry et al. have shown that amine functionalized CPBs could serve as chemosensors changing their optical properties (e.g., fluorescence) in variable chemical surroundings [30]. The synthesis of high grafting density CPBs could be achieved only by the methods compatible with the “grafting from” approach, such as surface-initiated Kumada catalyst transfer polymerization, surface-initiated Stille polycondensation, or self-templating surface-initiated polymerization (ST-SIP) [27]. The last technique is a two-step approach that firstly requires the synthesis of parent brushes (multimonomers) with pendant, e.g., thiophene or acetylene groups that can, in the next step, be converted into conjugated chains by another polymerization, resulting in the formation of ladder-like polymer brushes [31]. It is worth emphasizing that the parent brushes with pendant acetylene groups are also prone to convenient modification by click chemistry that allows the introduction of additional functionality to the brushes [32]. The synthesis of the parent brushes was so far realized by copper-based SI-ATRP [7] and surface-initiated photoiniferter-mediated polymerization (SI-PIMP) [33,34,35,36]. In the case of SI-ATRP, the developed synthetic conditions required the use of a high temperature and high concentration of copper-based catalyst [7]. SI-PIMP could be realized at room temperature, but high-energy UV radiation is needed to initiate the polymerization [34,35]. Furthermore, the formation of parent brushes requires the synthesis of bifunctional monomers, such as 3-methylthienyl methacrylate (MTM) and 3-trimethylsilyl-2-propynyl methacrylate (TPM), which are not commercially available; thus, the development of a synthetic strategy that enables an efficient decoration of flat surfaces in microliter volumes saving reactants is of great importance. 

In response to that, we present here a facile synthetic strategy, namely metal-free SI-ATRP, that allows the synthesis of surface-grafted polymers with pendant thiophene and acetylene groups using microliter volumes of reagents. The developed syntheses of poly (3-methylthienyl methacrylate) (PMTM) and poly (3-trimethylsilyl-2-propynyl methacrylate) (PTPM) brushes are triggered by visible light and could be performed under ambient conditions. Furthermore, for the first time, we present the formation of block copolymer brushes having both acetylene and thiophene pendant groups. Finally, the spatial control over the PMTM and PTPM brush growth is presented by utilizing easily accessible photomasks, such as TEM grids. 

## 2. Materials and Methods

### 2.1. Materials

ITO substrates (glass slides covered with 100 nm thick layer of ITO) were purchased from Ossila (Sheffield, UK). Silicon wafers were purchased from ON Semiconductor (Roznov, Czech Republic), while microscope cover slides were purchased from Equimed S.J. (Krakow, Poland). TEM square mesh support grids (nominal hole size 28 μm, material: nickel) were obtained from Micro to Nano (Haarlem, The Netherlands).

Toluene (p.a.), ethanol (p.a.), and chloroform (p.a.) were obtained from Chempur (Piekary Slaskie, Poland). 1,1,4,7,10,10-hexamethyltriethylenetetramine (HMTETA, 97%), copper (II) bromide (99.999%), copper (I) bromide (99.999%), triethylamine (TEA, ≥99.5%), N,N-dimethylformamide (DMF, HPLC, ≥99.9%), toluene (HPLC, GC, ≥99.9%), molecular sieves (4 Å), and 10-Phenylphenothiazine (PTH, ≥98%) were received from Sigma-Aldrich (Burlington, MA, USA). Iron (III) chloride (anhydrous, 98%) and bicyclo[2.2.1]hepta-2,5-diene-rhodium (I) chloride dimer was received from Alfa Aesar (Ward Hill, MA, USA). 3-(Trichlorosilyl)propyl 2-Bromo-2-methylpropanoate was purchased from TCI (Tokyo, Japan). N,N-Dimethylacetamide (DMA, anhydrous) was obtained from Fluorochem (Hadfield, UK). DMF, DMA, and toluene (HPLC, GC, ≥99.9%) were dried over molecular sieves before usage. The rest reagents were used as received. The monomers: 3-methylthienyl methacrylate (MTM) and 3-trimethylsilyl-2-propynyl methacrylate (TPM) were synthesized as described elsewhere [33,37].

### 2.2. Methods

FTIR spectra of PMTM and PTPM brushes were collected using FTIR spectrometer Nicolet iS10 (Thermo Scientific™, Waltham, MA, USA) with Grazing Angle Specular Reflectance Accessory. The measurements were performed using p-polarized light at the incident angle of 80°. All the spectra were averaged from 128 scans (scan resolution was set to 8 cm^−1^) and baseline corrected using OMNIC software. Atomic force microscopy (AFM) microphotographs were captured using the Dimension Icon AFM (Bruker, Santa Barbara, CA, USA) working in the PeakForce Tapping^®^ (PFT) and QNM^®^ modes with standard silicon cantilevers for measurements in air with a nominal spring constant of 0.4 N m^−1^. The thickness of the brushes was assessed by step height measurements as the difference between the polymer layer and uncovered inorganic substrate (the scratch was made using a needle). The average thickness of the polymer layer with uncertainty was calculated as an arithmetic mean of thicknesses measurements in a few different places. Images of TEM grids were obtained with a scanning electron microscope Phenom Pro (Model 800-07333, Phenom World, Eindhoven,The Netherlands) with the accelerating voltage set to 10 kV. Optical visualization of patterned polymer brushes (magnification 5×) was performed with an optical microscope Nikon Eclipse LV100 (Nikon Instruments Inc., Melville, NY, USA).

### 2.3. Procedures

#### 2.3.1. Surface Cleaning and Immobilization of the Initiator 

ITO and silicon substrates were first ultrasonicated in ethanol for 10 min and then treated for 30 min using UV ozone cleaning (UV-ozone cleaner with UV lamp dominant wavelengths: 185 and 254 nm, Ossila, Sheffield, UK). The immobilization of the trichlorosilane initiator was conducted according to the procedure reported by Saha and Baker [38]. The purified substrates were placed in toluene solution of 3-(trichlorosilyl)propyl 2-bromo-2-methylpropanoate (1 mM) and TEA (10 mM) under argon atmosphere for 24 h and then purified by rinsing with a copious amount of toluene and subsequent ultrasonication in toluene for 1 min. Such prepared initiator-decorated substrates were finally dried in the stream of argon.

#### 2.3.2. Surface-Initiated Atom Transfer Radical Polymerization (SI-ATRP) of 3-Methylthienyl Methacrylate (MTM) and 3-Trimethylsilyl-2-propynyl Methacrylate (TPM)

SI-ATRP was conducted following the slightly modified procedure reported previously by our team [7]. The molar ratio of reagents was as follows CuBr_2_ (1):CuBr (21):HMTETA (77):monomer (2000). The polymerizations were performed in the mixture of DMF (95%) and distilled water (5%). Initiator-functionalized ITO plates were used as substrates for the reactions. The polymerization time was set to 3 h and temperature to 70 °C for both MTM and TPM. Afterwards, the cleaning procedure included ultrasonication in DMF, THF, and toluene for 5 min and finally drying in the stream of argon. 

#### 2.3.3. Metal-Free Surface-Initiated Atom Transfer Radical Polymerization (Metal-Free SI-ATRP) of MTM and TPM

The homogenous reaction mixture for both monomers was prepared in the system of three amber vials sealed with rubber septa and connected via double-tipped needles. To ensure an oxygen-free atmosphere, the vials were purged with argon. Moreover, the first vial contained pure DMA to saturate the system with solvent vapors. The solution of monomer in DMA was placed in the second vial. After 10 min of argon purging, the monomer solution was transported via a double-tipped needle to the third vial with a solution of PTH in DMA. The resulting mixture was stirred on a magnetic stirrer for 5 min. PTH to monomer molar ratio was set to 1:100. Afterwards, 20 µL of the reaction solution was pipetted on the initiator-functionalized ITO substrate (size 15 × 15 mm) and then quickly covered by the cover glass (size 18 × 18 mm). Such prepared sample was placed below the homemade LED reactor (Figure S1, λ_max_ = 400 ± 5 nm, light intensity 18 ± 2 W/m^2^ measured by the Delta OHM HD2302.0 light meter equipped with the probe sensitive for the spectral range 400–1050 nm) under ambient conditions for a given time. After completion, the cover glass was removed, while the substrate with grafted polymer brushes was rinsed with DMA and toluene and subjected to impulse ultrasonication for 5 min (1 s of impulse and 1 s gap between impulses) in each solvent and finally dried in the steam of argon. The tested monomer concentrations were, respectively, 1 M and 2 M for MTM and 2.67 M for TPM. Moreover, the polymerization of MTM on the larger plate (ITO size 25 × 75 mm and cover glass size 40 × 90 mm, Figure S3) was carried out using 180 µL of reaction solution ([MTM] = 2 M, 1 mol% of PTH). The purification procedure was the same as described above.

#### 2.3.4. Chain Extension Experiment

At first, PMTM and/or PTPM brushes were grafted from ITO by means of metal-free SI-ATRP (time: 3 h, [MTM] = 2 M, [PTPM] = 2.67 M, 1 mol% of PTH) in DMA. In order to check the chain-end activity, PMTM and PTPM brushes were subjected to the second polymerization for 3 h using the same conditions as initially. The polymerization procedure and sample purification were the same as in Section 2.3.3.

#### 2.3.5. Synthesis of ITO-g-PMTM-b-PTPM and ITO-g-PTPM-b-PMTM Block Copolymer Brushes by Metal-Free SI-ATRP

PMTM and PTPM brushes were initially grafted on ITO using the same conditions as in Section 2.3.4. The obtained homopolymer PMTM and PTPM brushes were then subjected to metal-free SI-ATRP (time: 3 h) with the solution containing another monomer than used initially. After reaction completion, the substrate cleaning was conducted as mentioned before (Section 2.3.3.). As a result, ITO-g-PMTM-b-PTPM and ITO-g-PTPM-b-PMTM block copolymer brushes were obtained.

#### 2.3.6. Self-Templating Polymerization of ITO-g-PMTM-co-PTPM

The ITO-g-PMTM-co-PTPM brush was placed in the special holder in an amber vial containing 20 mg of FeCl_3_ with a magnetic stir bar. The vial was sealed with rubber septa and connected by a double-tipped needle with a vessel with 10 mL of distilled chloroform. Such prepared reaction system was placed in an ice bath and purged for 10 min with argon to get rid of oxygen. Afterwards, chloroform was transported to the vial with the plate. The reaction mixture was stirred for 1 h at 0 °C and then moved to the fridge for 24 h (7 °C, no stirring). After that time, the mixture was heated to room temperature and left to react for another 24 h without stirring. The sample was cleaned in the glove box under dark by intense rinsing with chloroform and methanol and finally dried in the stream of argon. Subsequent deprotection of pendant acetylene group and their polymerization using bicyclo[2.2.1]hepta-2,5-diene-rhodium(I) chloride dimer catalyst was performed as described elsewhere [34]. Each step of the copolymer conjugation was followed by FTIR measurements.

#### 2.3.7. Synthesis of Patterned Polymer Brushes by Metal-Free SI-ATRP

A few initiator-functionalized silicon plates (size 15 × 15 mm) were prepared. 20 µL of polymerization solution ([MTM] = 2 M, 1 mol% of PTH, solvent DMA) was pipetted on the silicon wafer and covered with glass (size 18 × 18 mm). To form the patterned polymer brushes, TEM grids were used to shield some spaces from LED radiation. Different arrangements of TEM grids were tested: on the top of a cover glass and under the cover glass on top of the initiator-decorated plate. Moreover, the influence of the TEM grid side on the quality of the formed patterned brushes was investigated. All the samples were polymerized for 4 h under the homemade LED reactor (λ_max_ = 400 nm) as described before. The cleaning procedure was the same as mentioned above (Section 2.3.3.).

## 3. Results

### 3.1. Copper-Based SI-ATRP

**Figure 1:**
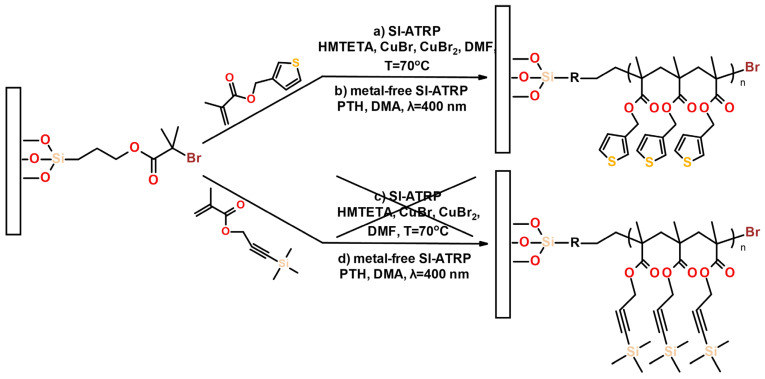
Scheme of a synthesis of PMTM and PTPM brushes via copper-based SI-ATRP and metal-free SI-ATRP.

**Figure 2:**
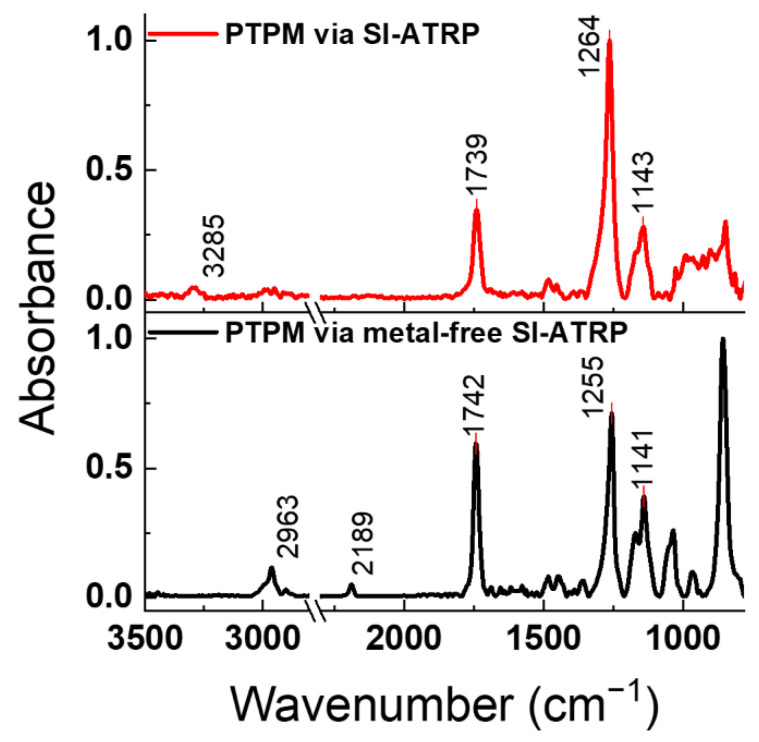
Normalized FTIR spectra of PTPM brushes synthesized via SI-ATRP and metal-free SI-ATRP. The spectra are normalized for clarity due to low absorbance intensity observed for the thin PTPM brushes prepared via classical SI-ATRP.

The “classical” copper-based SI-ATRP enables polymer brush formation via the grafting from approach. We have previously shown that it can be successfully used for the synthesis of PMTM brushes with thiophene pendant groups (Figure 1) [7]. However, when we tried to apply the SI-ATRP for the grafting of PTPM brushes (Figure 1), the obtained brushes were found to be very thin (about 4 nm), and the uncontrolled deprotection of the acetylene groups was observed (see below). The FTIR spectrum of the PTPM brushes after copper-based SI-ATRP (Figure 2) shows the characteristic band at 3285 cm^−1^ from unprotected acetylene groups (**H-C**≡C, C-H stretching vibrations) [33,34], while we could not observe the band around 2200 cm^−1^, characteristic for C≡C stretching vibrations in the protected acetylene group (Figure 2) [33,34]. The cleavage of protecting trimethylsilyl groups is promoted by the basic ATRP solution and high reaction temperature. This unwanted process leads to the partial polymerization of the acetylene groups and hinders the main chain propagation. Thus, one may conclude that the applied conditions of copper-based SI-ATRP are not suitable for the polymerization of the TPM monomer.

### 3.2. Metal-Free SI-ATRP of MTM and TPM Monomers

In order to find a facile synthetic strategy for obtaining both PTPM and PMTM brushes, we decided to test the metal-free SI-ATRP. Metal-free SI-ATRP enables the decoration of inorganic substrates by functional polymer brushes under ambient conditions using only microliter volumes of reagents [25,26], contrary to “classical” SI-ATRP that also often requires the utilization of a high temperature and unwanted copper compounds. PTH was chosen as an organic photocatalyst due to its desirable catalytic activity, availability, and high tolerance to oxygen [25]. This compound was earlier found to be able to absorb light from the visible range, showing highly reductive properties in the excited state [39]. That feature enables cleaving of the carbon-bromine bond and creating an organic radical center undergoing chain propagation, while the whole process is controlled through the reversible activation and deactivation of the growing active centers as described elsewhere [39].

**Figure 3:**
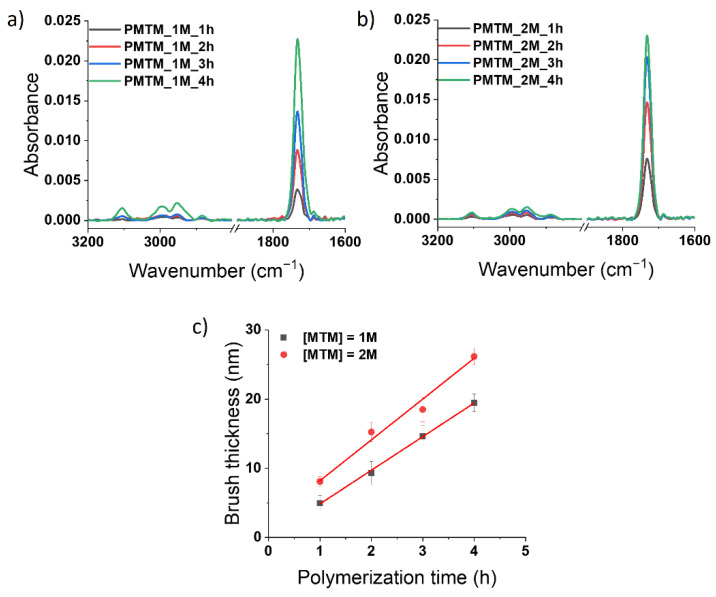
FTIR spectra of PMTM brushes captured after various times of metal-free SI-ATRP carried out using either 1 M (**a**) or 2 M (**b**) monomer concentration. The dependency of PMTM brush thickness (measured by AFM) vs. polymerization time (**c**).

**Figure 4:**
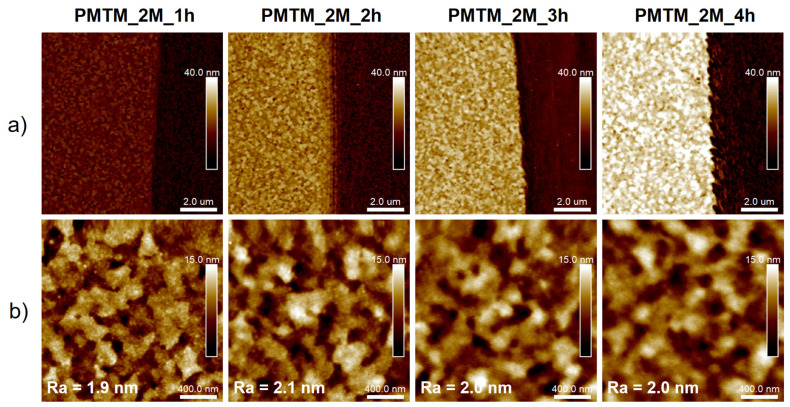
AFM topography images of PMTM brushes captured in air after different polymerization times at the edge of the scratch (**a**) and corresponding high-resolution images (**b**) with average roughness (Ra) values. Note that AFM images are presented on the same height scale.

At first, we attempted to obtain PMTM brushes via metal-free SI-ATRP on ITO substrates (Figure 1). The influence of the monomer concentration and irradiation time on the brush growth were tested. It was indicated that PMTM brushes obtained using a higher concentration of MTM ([MTM] = 2 M; abbreviated as PMTM_2M_time (h)) were thicker than analogue brushes grafted using 1 M solution of the monomer (abbreviated as PMTM_1M_time (h)). The received PMTM brushes were characterized by FTIR spectroscopy and AFM. The analysis of the most characteristic IR bands (Figure 3a,b) confirms the formation of PMTM brushes (e.g., band at 3105 cm^−1^ C-H stretching vibration in thiophene ring, 2998 cm^−1^ and 2938 cm^−1^ C-H stretching vibration in alkyl groups, 1731 cm^−1^ C=O stretching vibration in ester group) [7,35]. Due to the usage of ITO plates serving as a mirror for the infrared radiation, the absorbance changes proportionally to the thickness of the polymer layer. As a result, for both the MTM concentrations (see Figure 3a,b), the increase in the absorbance of the IR bands (see especially very intense IR band at 1731 cm^−1^) with the increase in polymerization time were observed. The comparison of the absorbance values of the band at 1731 cm^−1^ (C=O stretching in the ester group) qualitatively supports the observed thickness increase of the brushes as revealed from the AFM measurements (Figure 3c and Figure 4). As PMTM brushes were synthesized without the presence of a sacrificial initiator, the linear dependency of brush thickness versus polymerization time was observed for both concentrations of MTM monomer in the range of 1 to 4 h of polymerization time (Figure 3c), similar to what was reported for controlled metal-free SI-ATRP of methyl methacrylate [24] and 2-(dimethylamine)ethyl methacrylate [25]. However, the deviation from the linear growth was noticed for longer reaction times due to the slowing down of the polymerization. The maximum PMTM brush thickness of about 44 nm was achieved when the reaction was conducted for 24 h (PMTM_2M_24h) (see Figure S2). Owing to the simplicity of the reaction system in metal-free SI-ATRP, this procedure was successfully applied to decoration of a larger ITO plate, resulting in the formation of a continuous polymer layer on the whole plate (Figure S3). It creates an opportunity for the facile modification of large area substrates using only microliter volumes of reagents (ca. 10 μL/cm^2^).

The AFM images of the PMTM brushes captured at the edge of the scratch show a gradual increase in the brush thickness with polymerization time (Figure 4a and Figure S4). The obtained high-resolution topography maps (Figure 4b) confirm the formation of a homogeneous layer on the ITO substrate. It is worth emphasizing that the brushes are characterized by low roughness parameters (average roughness R_a_~2 nm) that are virtually the same for each sample (Figure 4b).

**Figure 5:**
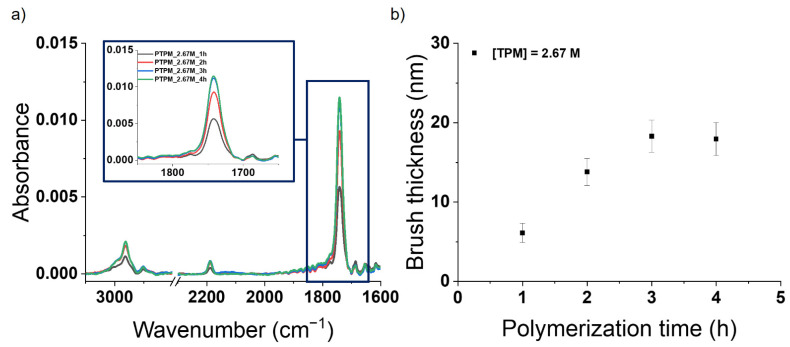
**(a)** FTIR spectra of PTPM brushes obtained after various times of metal-free SI-ATRP with the zoomed range corresponding to the absorbance of carbonyl group and (**b**) dependence of brush thickness (measured by AFM) vs. polymerization time.

**Figure 6:**
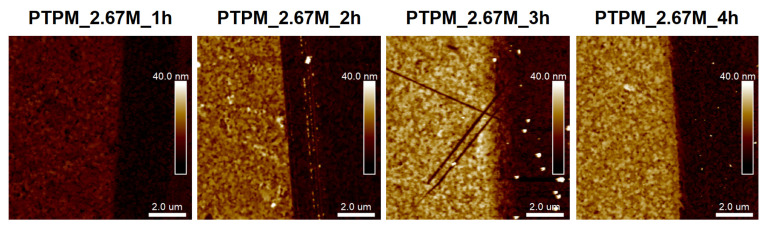
AFM images captured at the edge of the scratch of PTPM brushes obtained after different times of metal-free SI-ATRP. Note that AFM images are presented on the same height scale.

Due to the unsuccessful grafting of PTPM brushes via copper-based SI-ATRP, the metal-free methodology was used. The utilization of this approach for the polymerization of the TPM monomer with the acetylene group was much more successful and allowed for the formation of PTPM brushes with various thicknesses. The kinetic investigation for the selected monomer concentration ([TPM] = 2.67 M) was performed (see Figure 5). The FTIR spectrum of the PTPM brushes shows characteristic bands at 2962 cm^−1^ (C-H stretching vibrations in alkyl groups), 2187 cm^−1^ (C≡C stretching vibrations), and 1741 cm^−1^ (C=O stretching vibrations in ester groups), confirming the structure of the brushes [34] and the presence of the protected acetylene groups in contrast to copper-based SI-ATRP (Figure 2). The AFM measurements revealed linear brush growth for short polymerization times (1–3 h, as presented in Figure 5b). For the polymerization time longer than 3 h, no further brush growth could be observed (see Figure 5b). It is clearly visible on the AFM images showing practically the same thickness after 3 and 4 h of polymerization (Figure 6), as well as the FTIR spectra (Figure 5a) as the absorbances are virtually the same for the brushes obtained after these times.

### 3.3. Chain Extension and Formation of Block Copolymer Brushes

**Figure 7:**
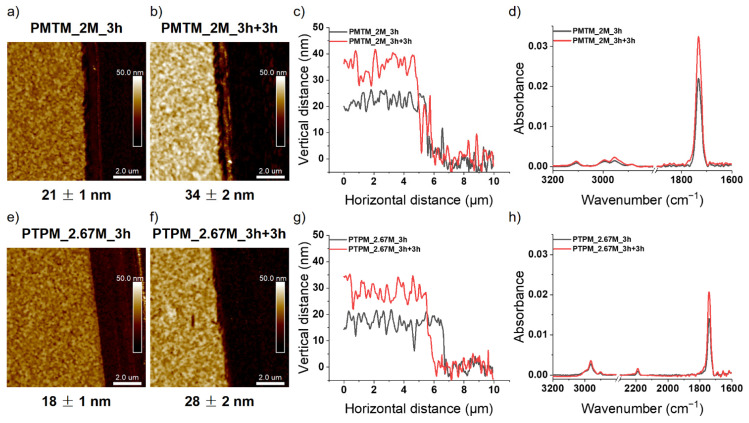
AFM images with representative cross-section profiles and FTIR spectra captured before and after chain extension of PMTM (**a**–**d**) and PTPM brushes (**e**–**h**).

In order to check the activity of the chain ends, PMTM_2M_3h and PTPM_2.67M_3h were subjected to the second polymerization using the same conditions (the samples will be abbreviated as PMTM_2M_3h+3h and PTPM_2.67M_3h+3h). Comparing the AFM images of the PMTM brush before and after the second polymerization (Figure 7a,b), one can notice a significant increase in the polymer layer thickness from 21 nm to 34 nm (Figure 7a–c). The percentage increase could be estimated at 62%, which is in good agreement with the calculated percentage increase (51%) of absorbance from the FTIR spectra (Figure 7d). The similar effect was observed for the PTPM brush. After 3 h of additional polymerization, the thickness of the brushes increased from 18 nm to 28 nm (Figure 7e,f), which equals to a 56% increase (Figure 7g). Based on the presented results for the extended polymer chains, we indicated that, for both brushes, the activity of the chain ends seems to be maintained after finishing the first polymerization, which enables the further elongation of the polymer chains. Therefore, the observed exemption of polymerization in the case of the PTPM brushes after 3 h is likely not associated with the irreversible termination of the polymer chains. One may rather expect the occurrence of some other side reactions that could hinder the brush growth.

**Figure 8:**
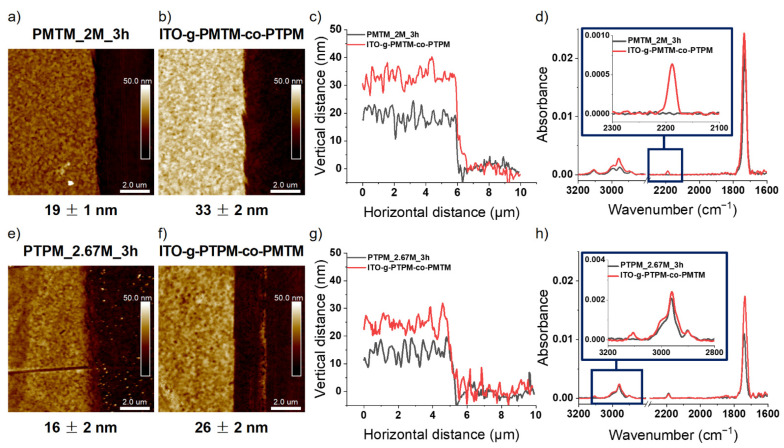
AFM images and cross-section profiles of: PMTM_2M_3h (**a**,**c**), ITO-g-PMTM-co-PTPM (**b**,**c**), PTPM_2.67M_3h (**e**,**g**), and ITO-g-PTPM-co-PMTM brushes (**f**,**g**). FTIR spectra of: (**d**) PMTM_2M_3h and ITO-g-PMTM-co-PTPM brushes, as well as (**h**) PTPM_2.67M_3h and ITO-g-PTPM-co-PMTM brushes.

Having confirmed the activity of the chain ends, we synthesized the block copolymer brushes by the sequential metal-free SI-ATRP of two different monomers on the same substrate. The applied polymerization procedures were analogue as for the homopolymer brushes. All the polymerizations were carried out for 3 h in the same conditions using the following concentrations of monomers [MTM] = 2 M and [TPM] = 2.67 M. We synthesized the block copolymer brushes in which the first block (close to the substrate) was either PMTM (will be abbreviated as ITO-g-PMTM-co-PTPM) or PTPM (ITO-g-PTPM-co-PMTM). The AFM measurements revealed an increase of the brush thicknesses from 19 nm (homopolymer PMTM) to 33 nm for ITO-g-PMTM-co-PTPM (Figure 8a–c) and from 16 nm (homopolymer PTPM) to 26 nm for ITO-g-PTPM-co-PMTM (Figure 8e–g). Any domain or phase separations were not observed, and the obtained layers were uniform on the whole surface.

In order to confirm the presence of two chemically different segments of the polymer chain, the FTIR spectra were collected. The identification of a new band at 2187 cm^−1^ for ITO-g-PMTM-co-PTPM (Figure 8d) after the second polymerization confirmed the presence of protected acetylene groups characteristic of the PTPM chain [34] and, hence, the formation of block copolymer brushes. Furthermore, an increase in the signal from C-H stretching (2960 cm^−1^) and C=O stretching vibrations (1734 cm^−1^) provided additional evidence of the successfulness of the synthesis. In the case of the ITO-g-PTPM-co-PMTM formation of the block copolymer brush, after, the second polymerization was indicated by the appearance of a new band at 3105 cm^−1^, which had been not observed initially and came from C-H stretching vibrations in the thiophene ring (Figure 8h) [35]. An increase in the absorbance of the IR bands assigned to the carbonyl and alkyl main chain groups additionally confirmed the formation of the expected structure. These results clearly show that the developed conditions enable the synthesis of advanced polymer structures with desirable functionality as both thiophene and acetylene groups could be easily converted into conjugated chains (see below). Furthermore, acetylene groups are prone to further modification, e.g., by click chemistry [32].

### 3.4. Polymer Chain Conjugation

**Figure 9:**
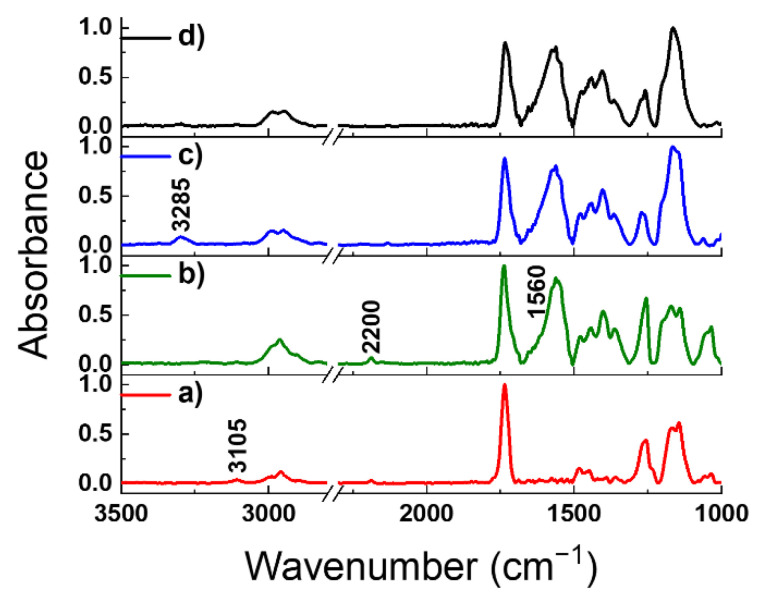
The comparison of normalized absorbance FTIR spectra of ITO-g-PMTM-co-PTPM brushes after metal-free SI-ATRP (**a**), oxidative polymerization of thiophene groups using FeCl_3_ (**b**), acetylene group deprotection (**c**), and polymerization of pendant acetylene using Rh-based catalyst (**d**).

The generation of conjugated polythiophene and polyacetylene chains within the block copolymer brushes was realized by self-templating surface-initiated polymerization (ST-SIP). The oxidative polymerization of thiophene groups in the presence of FeCl_3_ (PMTM) and polymerization of deprotected acetylene groups catalyzed by bicyclo[2.2.1]hepta-2,5-diene-rhodium(I) chloride dimer (PTPM) were previously described as efficient approaches for obtaining conjugated PMTM and PTPM brushes [33,35]. Therefore, we used these previously developed procedures to form conjugated block copolymer brushes ITO-g-PMTM-co-PTPM having both polythiophene and polyacetylene chains in the structure. At first, the oxidative polymerization of the thiophene groups in the PMTM chains catalyzed by FeCl_3_ was conducted in the dry chloroform. As a result, almost the complete disappearance of the band at 3105 cm^−1^ from C-H stretching vibrations in the thiophene ring and the appearance of a strong band at ca. 1560 cm^−1^ from C=C stretching vibration in polythiophene chains [35,40] (Figure 9b) were observed, confirming the successful conversion of thiophene groups into conjugated chains. In the next step, pedant acetylene groups from the PTPM outer chains were deprotected using a saturated solution of K_2_CO_3_ in methanol and THF [34]. In consequence, the appearance of the characteristic band at 3285 cm^−1^ (from C-H stretching vibrations in H-C≡C) was observed (Figure 9c) due to the cleavage of the trimethylsilyl group. Finally, the ST-SIP polymerization of acetylene groups catalyzed by bicyclo[2.2.1]hepta-2,5-diene-rhodium(I) chloride dimer was confirmed by the almost complete disappearance of the band at 3285 cm^−1^ (Figure 9d), characteristic of the deprotected acetylene groups. These preliminary experiments indicate the possibility of the creation of advanced polymer architectures for the directional transport of electrons and excitation energy.

### 3.5. Spatially Controlled Decoration of Inorganic Substrates

The mechanism of metal-free SI-ATRP is based on the process initiated by radiation, so spaces that are shielded from light exposure during polymerization are expected to remain uncovered with a polymer layer. In the conducted experiments, the TEM grids were used as „photomasks” to create patterned PMTM brushes on silicon wafers. We have tested two TEM grid locations. The placement of the grid on the cover glass resulted in obtaining only an outline of it within the polymer layer (which was visible on the optical microscope as a blurry shape, Figure S5c). Due to low spatial resolution, we did not observe the formation of patterned films. However, when the grid was placed under the cover glass, significantly higher resolution was achieved, as shown on optical microscope images (see Figure S5d).

**Figure 10:**
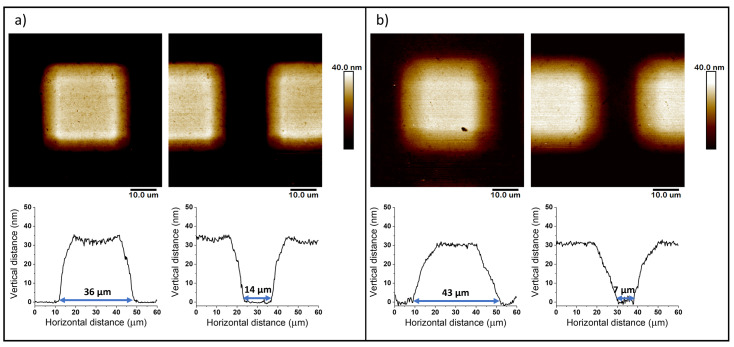
AFM images with corresponding cross-section profiles of patterned PMTM brushes synthesized by metal-free SI-ATRP by placement of the TEM grid with (**a**) shiny side faced to LED lamps and (**b**) matte side faced to LED lamps.

The AFM characterization of the patterned PMTM brushes also allowed the identification of the difference between the polymerizations with the TEM grid facing different sides to the light. One grid position (with the shiny side faced to the LED lamps) gave the pattern with sharp edges of squares (square diameter = 36 μm) and well-visible spaces between neighboring squares (gap size = 14 μm) (Figure 10a). The obtained dimensions of the created PMTM patterns slightly vary from its real size (see SEM measurements of TEM grid, Figure S6) by about a few micrometers. It may be explained by the convolution of the AFM tip shape in the obtained images.

The second grid position (with the matte side faced to the LED lamps) provided worse spatial control and fuzzy square edges (square diameter = 43 μm and gap size = 7 μm, Figure 10b). In order to explain the AFM results, both sides of the TEM grid were analyzed by the SEM. It turned out that the TEM grid edges on the matte side were straight and sharp (Figure S6a), while the shiny side had an additional frame around every square (Figure S6b,c). When the polymerization was carried out with the matte side faced to the LED lamps, the radiation could have been refracted on the fault, and the final brush pattern was blurrier at the edges. Therefore, the observed less-steep edges in Figure 10b could be assigned to proceeded photopolymerization, even partially underneath the grid due to light scattering.

**Figure 11:**
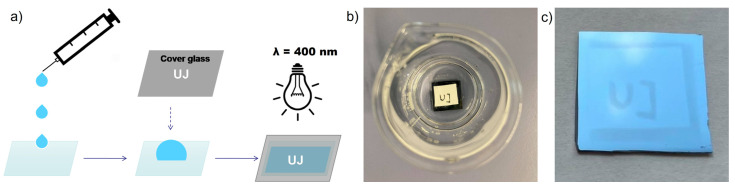
Scheme of the synthetic approach used for the creation of “UJ” pattern (**a**) with photos presenting plate with white homemade photomask (**b**) and final PTPM brushes grafted from silicon wafers (**c**).

In order to present the ease of the developed method, we prepared a homemade photomask made of sticky foil with the inscription “UJ”, which states the Polish abbreviation of Jagiellonian University (Figure 11a,b). The foil was placed on the glass slide used to cover the initiator decorated substrate with polymerization solution. The 4-h polymerization enabled to obtain the pattern visible by the naked eye (Figure 11c). According to the presented results, it is expected that a much more complicated pattern could be formed depending on the used photomask.

## 4. Conclusions

In summary, we presented here the utilization of metal-free SI-ATRP for the efficient decoration of flat inorganic substrates by functional surface-grafted polymers bearing acetylene and thiophene groups. PTPM brushes that are difficult to produce by means of “classical” SI-ATRP were successfully prepared here, omitting the problems with the unintentional deprotection of the acetylene groups. The utilization of metal-free SI-ATRP in the case of the synthesis of both PTPM and PMTM brushes enables the reduction in temperature and the reaction mixture volume when compared to copper-based SI-ATRP. The revealed linear growth of the brushes enables the control and adjustment of the layer thickness by the simple manipulation of the monomer concentration and polymerization time. Moreover, the obtained brushes are characterized by high chain-end fidelity. Metal-free SI-ATRP allowed obtaining block copolymer brushes having both pendant thiophene and acetylene groups, which could be easily converted into conjugated brushes by sequential self-templating polymerization. The low consumption of chemicals and exclusion of often unwanted copper-based catalysts make this method economical and environmentally friendly. The developed methodology provides a powerful solution for non-experts for the production of complex polymer structures, such as block copolymer, as well as patterned brushes potentially important in regard to certain optoelectrical applications, such as sensors, photovoltaic solar cells, or nanoelectronics. It also opens the path for scaling up the formation of the brushes, including the patterned conjugated ones.

## Data Availability

Data are contained within the article or Supplementary Material.

## Supplementary Materials

The following are available online at https://www.mdpi.com/article/10.3390/polym13244458/s1, Figure S1: Photos of the homemade LED reactor, Figure S2: AFM topography image of PMTM_2M_24h brush at the edge of the scratch (a) with representative cross-section profile (b), Figure S3: The photo of the PMTM_2M_4h brushes grafted from the large area ITO substrate, Figure S4: AFM topography images of PMTM brushes captured after different polymerization times at the edge of the scratch (a) with corresponding high-resolution images (b) with an average roughness (Ra) value, Figure S5: Schemes of the two approaches used for obtaining patterned PMTM brushes by metal-free SI-ATRP: with the TEM grid on top of (a) or under (b) cover glass. Optical microscope images of PMTM brushes obtained by placing the TEM grid on top of (c) or under (d) cover glass, Figure S6: SEM images of (a) matte and (b,c) shiny side of the TEM grid.
