# Supplementary material for: Preparation of Homopolymer, Block Copolymer, and Patterned Brushes Bearing Thiophene and Acetylene Groups Using Microliter Volumes of Reaction Mixtures
Source: Polymers (Basel). 2021 Dec 19;13(24):4458. doi: 10.3390/polym13244458 (PMC8704565; doi:10.3390/polym13244458)
Supplement: Supplementary file 1 [file polymers-13-04458-s001.zip › polymers-1494271-supplementary.pdf]

Supporting Information

Preparation of homopolymer, block copolymer and patterned brushes bearing thiophene and acetylene groups using microliter volumes of reaction mixtures

Joanna Smenda[1], Karol Wolski[1]*, Kamila Chajec[1], Szczepan Zapotoczny[1]

1 Faculty of Chemistry, Jagiellonian University, Gronostajowa 2, 30-387 Krakow, Poland; joanna.smenda@doctoral.uj.edu.pl (J.S.), wolski@chemia.uj.edu.pl (K.W.), kamila.chajec@doctoral.uj.edu.pl (K.C.), zapotocz@chemia.uj.edu.pl (S.Z.)

* Correspondence: wolski@chemia.uj.edu.pl

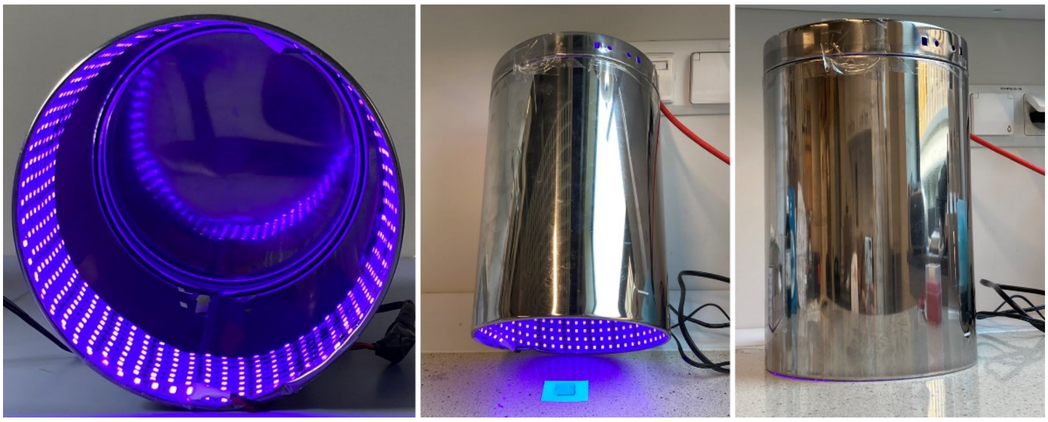

Figure S1. Photos of the homemade LED reactor.

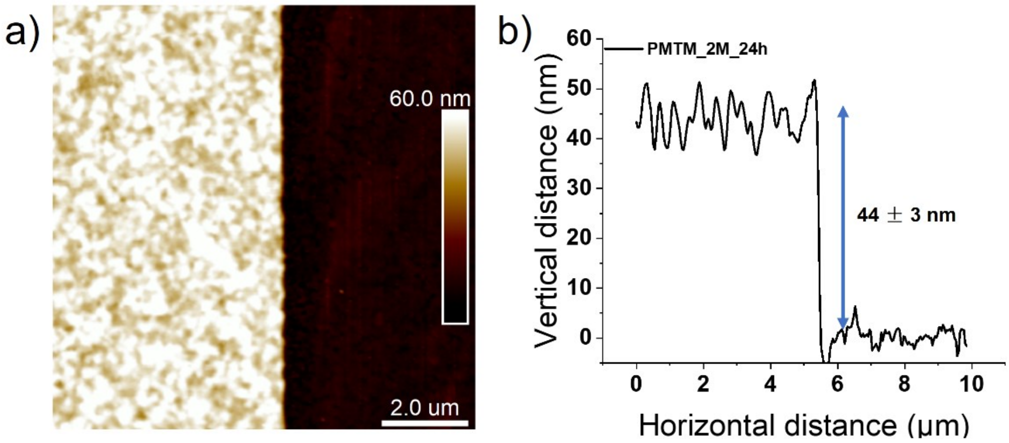

Figure S2. AFM topography image of PMTM_2M_24h brush at the edge of the scratch (a) with representative cross-section profile (b).

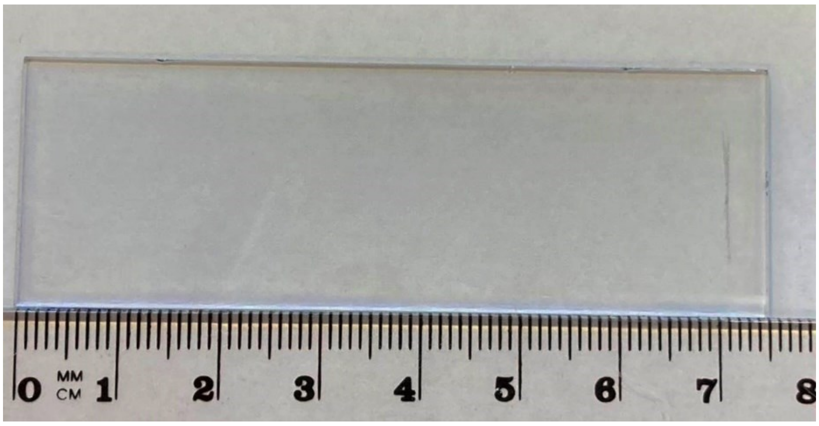

Figure S3. The photo of the PMTM_2M_4h brushes grafted from the large area ITO substrate.

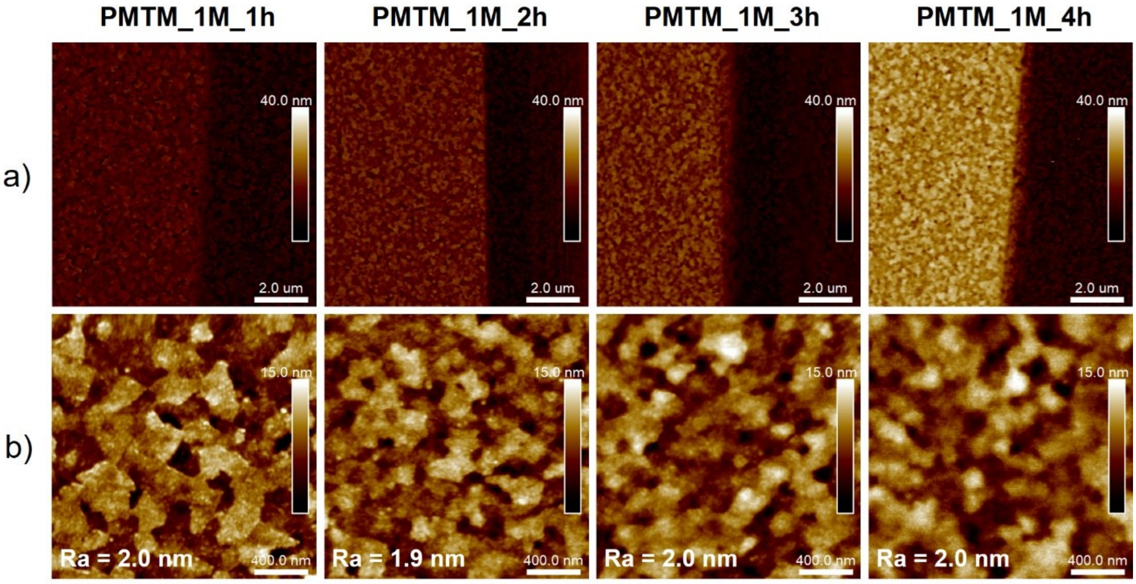

Figure S4. AFM topography images of PMTM brushes captured after different polymerization times at the edge of the scratch (a) with corresponding high-resolution images (b) with average roughness (Ra) values. Note that AFM images are presented on the same height scale.

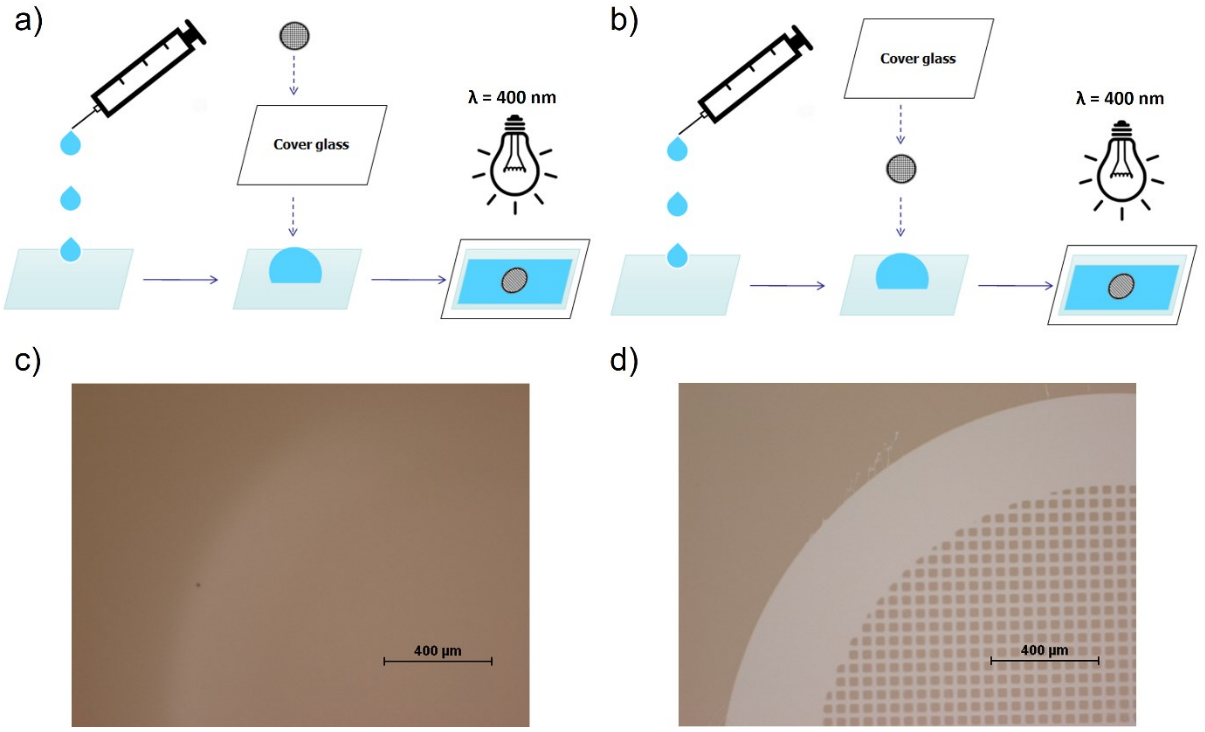

Figure S5. Schemes of the two approaches used for obtaining patterned PMTM brushes by metal-free SI-ATRP: with the TEM grid on top of (a) or under (b) cover glass. Optical microscope images of PMTM brushes obtained by placing the TEM grid on top of (c) or under (d) cover glass.

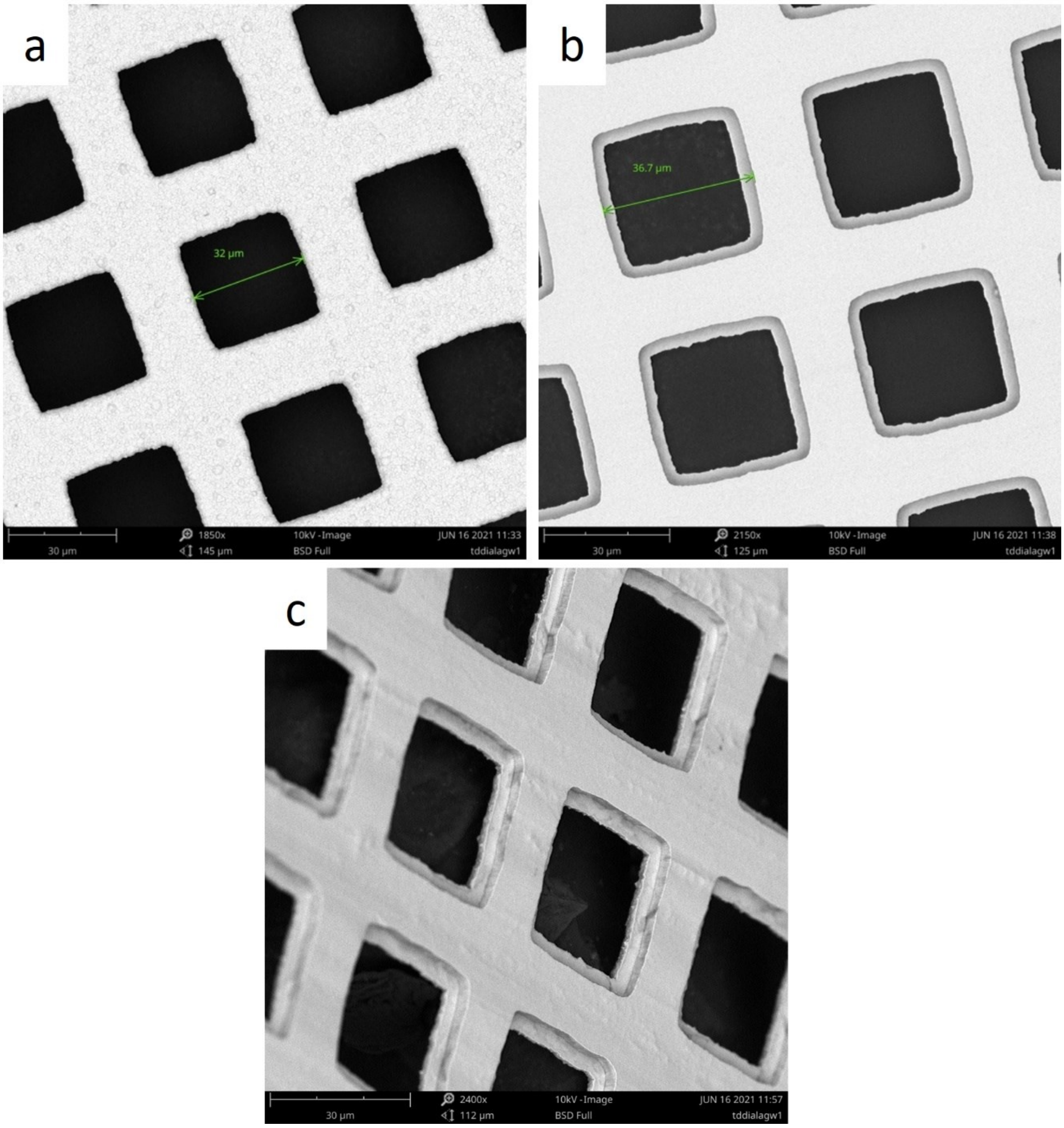

Figure S6. SEM images of a) mate and b), c) shiny side of the TEM grid.
